# Detection of PHLPP1α/β in Human and Mouse Brain by Different Anti-PHLPP1 Antibodies

**DOI:** 10.1038/srep09377

**Published:** 2015-04-01

**Authors:** Travis C. Jackson, Hülya Bayir, Milos D. Ikonomovic, Keri Janesko-Feldman, Zaichuan Mi, Tianyan Gao, Edwin K. Jackson, Patrick M. Kochanek

**Affiliations:** 1 University of Pittsburgh School of Medicine, Department of Critical Care Medicine, Safar Center for Resuscitation Research, 200 Hill Building, 3434 Fifth Avenue; 2 University of Pittsburgh School of Medicine, Department of Pharmacology and Chemical Biology, Bridgeside Point Building 1, 100 Technology Drive; 3 University of Pittsburgh School of Medicine, Department of Neurology, 811 Kaufmann Medical Building, 3471 Fifth Avenue; 4 University of Kentucky College of Medicine, Department of Molecular & Cellular Biochemistry, Markey Cancer Center, Lexington, KY 40536, USA

## Abstract

Pleckstrin homology domain and leucine rich repeat protein phosphatase 1 (PHLPP1) is a member of the serine/threonine family of phosphatases. It has been studied in organs including brain, heart, pancreas, adipose, breast, and prostate. Human PHLPP1 encodes two splice variants - PHLPP1α (~140–150 kDa) and PHLPP1β (~180–190 kDa). Commercial antibodies are widely used to characterize PHLPP1 proteins in cells/tissues. Here we validate five different antibodies to detect PHLPP1α/β by Western blot using PHLPP1 WT/KO mice. All antibodies recognize PHLPP1β in brain. Only a single antibody (Cosmo Bio Co) detects PHLPP1α (~145–150 kDa). The other four antibodies detect a non-specific signal at ~150 kDa as evidenced by its abundance in PHLPP1 KO tissues. Results suggest Cosmo antibody is a better reagent to detect PHLPP1α by Western blot. In contrast, we found it unsuitable for immunofluorescence applications in brain. Our findings caution interpretation of the ~150 kDa band detected by some PHLPP1 antibodies in rodent and human tissues. Results also recapitulate the importance of including molecular weight standards in Western blot data to simplify retrospective analysis.

Pleckstrin homology domain and leucine rich repeat protein phosphatase 1 (PHLPP1) is a serine/threonine phosphatase with two splice variants. PHLPP1β was first discovered in rat brain^1^. Neuronal PHLPP1β strongly inhibits extracellular regulated kinase (ERK) activation^2^,^3^, and regulates hippocampal dependent memory in mice^4^. PHLPP1α was later cloned from a human cDNA collection^5^. PHLPP1α overexpression in cancer cells potently inhibits pro-survival molecule protein kinase B (AKT) and induces cell death^5^.

Immuno-based techniques such as Western blot are routinely used to study PHLPP1 in cell death and disease. The first studies characterizing endogenous PHLPP1α by Western blot reported a ~140 kDa migrating protein on SDS-PAGE, using a custom made antibody^6^. Likewise, we reported a ~140 kDa PHLPP1α protein in rat brain homogenates using a goat polyclonal antibody obtained from Santa Cruz Biotechnology^7^. Later we reported a PHLPP1α protein migrating at ~145–150 kDa in rat brain/neuron culture homogenates using a rabbit polyclonal antibody purchased from Bethyl laboratories^3^. The authenticity of endogenous rat ~150 kDa PHLPP1α was checked by overexpression studies in human embryonic kidney (HEK) cells. We identified a dominant ~150 kDa band only in HEKs overexpressing human PHLPP1α^3^. Those results led us to speculate that minor differences observed in PHLPP1α molecular weight (+/− 5 kDa) across studies was likely caused by post-transcriptional modifications or slight adjustments in experimental factors (e.g. % SDS-PAGE gel used for sample electrophoresis).

Here we used PHLPP1 gene knockout (KO) mice to unambiguously authenticate target specificity of several common, as well as seldom used, PHLPP1 antibodies. Gene deletion in KO mice was confirmed by Western analysis of PHLPP1 in tissue extracts. All antibodies detected the larger ~190 kDa PHLPP1β variant, as confirmed by its absence in KO mice. A presumed ~150 kDa PHLPP1α signal was detected by four of the antibodies but equally present in PHLPP1 KO mice – indicating it is likely a non-specific signal. A fifth relatively new polyclonal commercial antibody uniquely recognized a ~145–150 kDa PHLPP1α signal absent in KO mice. Our findings suggest that antibody selection is a key experimental factor in the study of PHLPP1α (particularly in brain).

## Results

**Figure 1:**
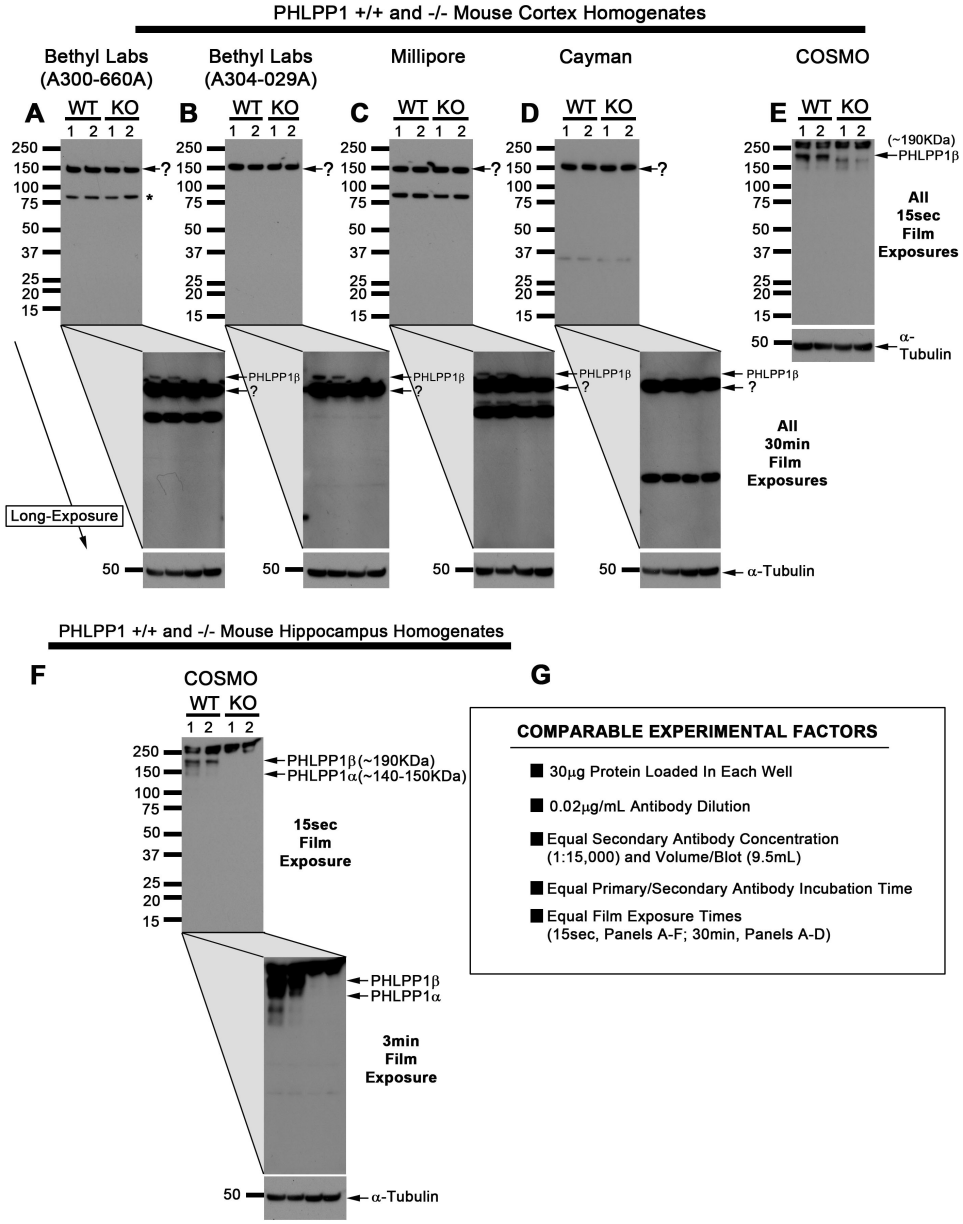
PHLPP1 Antibody Comparison in WT and KO Brain. Cortex and Hippocampus were harvested from PHLPP1 WT/KO mice. 30 μg/well homogenized brain tissue extract was loaded onto 15 well/4–15% gradient SDS-PAGE gels. PVDF membranes were probed with PHLPP1 antibodies purchased from (A) Bethyl Laboratories A300-660A, (B) Bethyl Laboratories A304-029A, (C) Millipore, (D) Cayman Chemical, and (E and F) Cosmo Bio Co Ltd. Two film exposure times were collected (TOP images show short film exposure and BOTTOM images show long film exposures of the same blot). (G) Experimental factors were controlled between antibodies to allow direct comparison of specificity and selectivity. Question mark (?) indicates a ~150 kDa band currently presumed to be PHLPP1α but is abundant in PHLPP1 KO mice. All blots show n = 2/genotype.

Brain tissue was harvested from PHLPP1 KO and WT mice. Total protein extracts were probed with anti-PHLPP1 antibodies (Figure 1). Antibodies obtained from Bethyl Laboratories (Figure 1A and 1B), Millipore (Figure 1C), and Cayman Chemical (Figure 1D) all detected a dominant band in WT mice with apparent molecular mass of ~150 kDa after 15 s film exposure. The ~150 kDa band is presumed to be PHLPP1α. However, contrary to that assumption, we observed equal levels in KO mouse cortex (Figure 1A–1D-Top Panels).

The PHLPP1β variant was clearly visible after 30 min film exposure using most antibodies (still faint using the Cayman antibody), and absent in KO extracts (Figure 1A–1D – Lower Panels). A second dominant ~90 kDa signal was also detected in Bethyl Laboratories antibody (A300-660A; Figure 1A - asterisk) and Millipore antibody which has been identified as β-catenin^8^. β-catenin was not detected by Bethyl Laboratories antibody (A304-029A; Figure 1B) or Cayman antibody (Figure 1D). PHLPP1β was readily detected by Cosmo antibody after 15s film exposure. Cosmo did not detect the ~150 kDa non-specific band (Figure 1E). In addition, it detected a strong PHLPP1α signal in WT hippocampal extracts as evidenced by its absence in KOs (Figure 1F). Key experimental factors were controlled for all antibody comparisons (Figure 1G).

**Figure 2:**
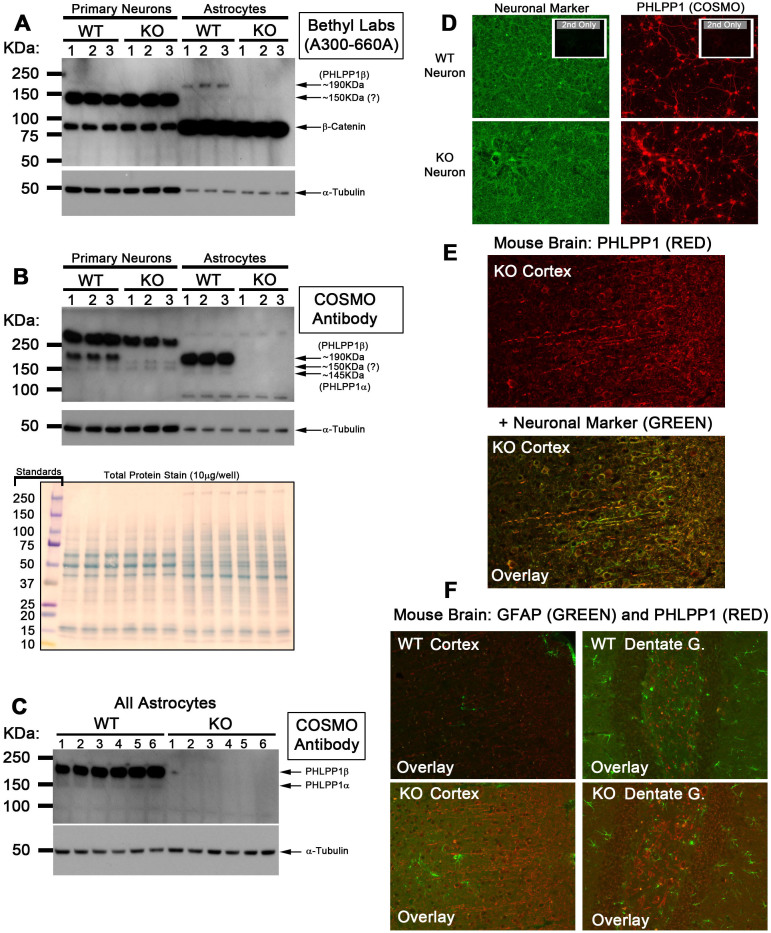
Utility of Cosmo Antibody for Western and Immunofluorescence Applications. WT/KO culture homogenates were prepared from enriched primary neurons and astrocytes and loaded onto SDS-PAGE gels (10 μg/well; n = 3/group). (A) PVDF membranes were probed with Bethyl laboratories antibody A300-660A (B) PVDF membranes were probed with Cosmo antibody. Protein loading control differences (i.e. α-tubulin) are not caused by loading control errors. Rather, differences in cell type as verified by total protein stain. (C) Western blot confirming detection of PHLPP1α in KO astrocyte homogenates probed with the Cosmo antibody (20 µg/well; n = 6/group). (D) 40× images showing DIV8 cultured WT/KO neurons stained with neuronal β tubulin III (neuronal marker; GREEN) and Cosmo PHLPP1 antibody (RED). Small boxes in upper right corner show respective secondary only controls. Little to no staining was observed. (E) 20× images of cortex in PHLPP1 KO stained neuronal β tubulin III (GREEN) and Cosmo PHLPP1 antibody (RED). Top image shows RED fluorescence only. Bottom image shows RED/GREEN overlay. (F) 20× images of cortex and dentate gyrus in PHLPP1 WT/KOs stained with GFAP (astrocytes; GREEN) and Cosmo PHLPP1 antibody (RED). Overlays show RED fluorescence does not co-localize with GREEN fluorescence.

We next evaluated the impact of antibody selection by testing if scientific conclusions change by reagent. Protein expression levels of PHLPP1α/β in neurons versus astroctyes were investigated. Performing the experiment with Bethyl laboratories antibody (A300-660A) revealed that “PHLPP1α” (i.e. ~150 kDa band) is highly expressed in neurons and absent in astrocytes (Figure 2A). Repeating the experiment with the Cosmo antibody gave different results - PHLPP1β is the only variant expressed in neurons. PHLPP1α and PHLPP1β are both expressed in astrocytes (Figure 2B and 2C).

Immunohistochemistry (IHC) is commonly used to define distribution/localization patterns of antigens in cells and tissues. The Cosmo antibody appears to be a superior reagent to detect PHLPP1α/β by Western analysis. We tested Cosmos's suitability for IHC applications in brain. A strong fluorescent signal (RED) was observed in both cultured primary WT and KO neurons (Figure 2D). Consistent with those findings, the Cosmo antibody gave strong fluorescence (RED) which co-localized with neuronal marker (GREEN) in cortical brain sections (Figure 2E – Lower panel shows YELLOW overlay). Finally, Cosmo staining (RED) was observed in both WT/KO mouse brain but failed to co-localize with astrocyte marker GFAP (Figure 2F).

**Figure 3:**
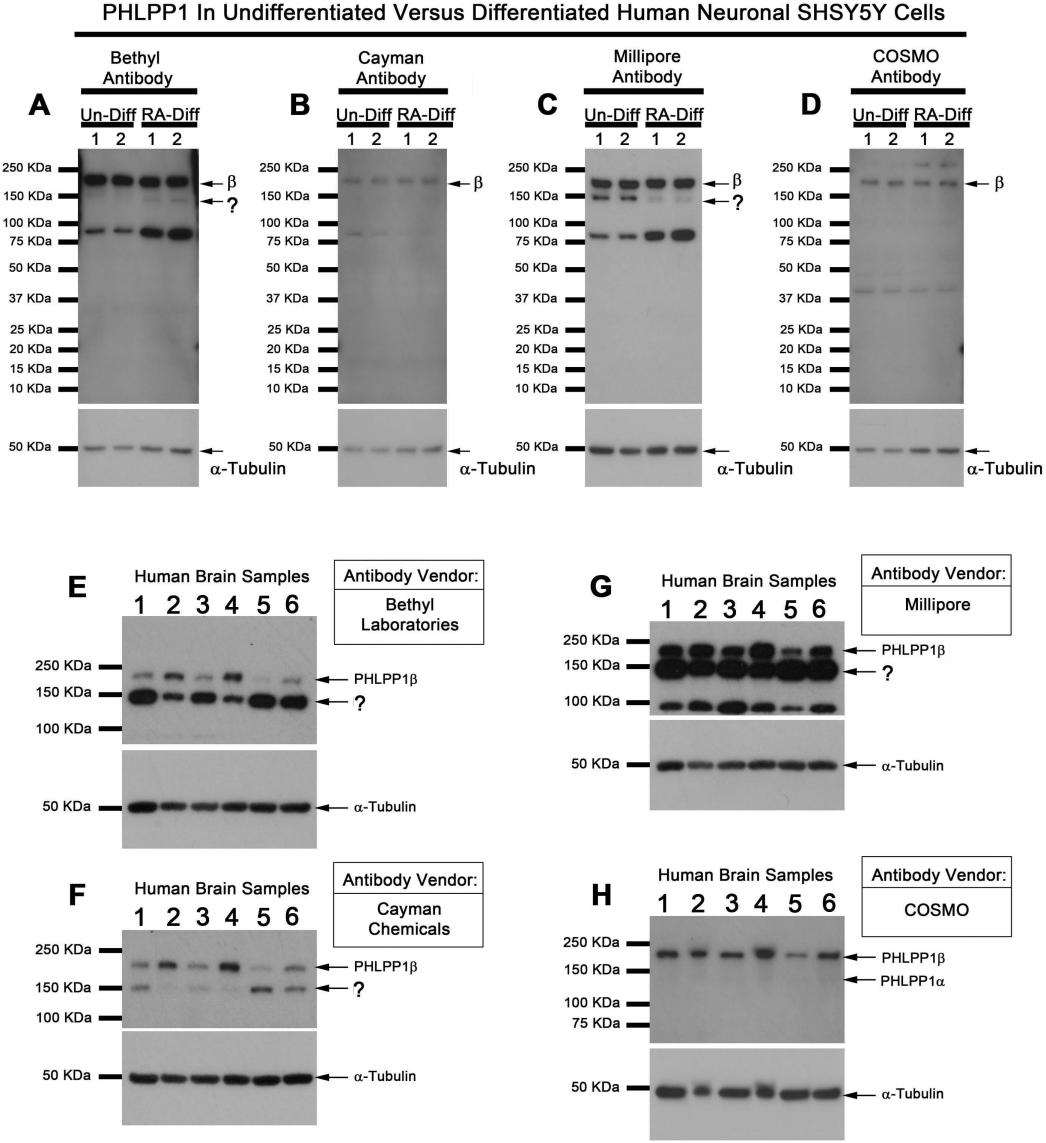
Detection of PHLPP1 Variants in Human Neuronal cells and Brain Tissue. Undifferentiated and differentiated SHSY5Y extracts (20 μg/well; n = 2) were probed with (A) Bethyl Laboratories A300-660A, (B) Cayman Chemical, (C) Millipore, and (D) Cosmo Bio Co Ltd. Anti-PHLPP1 antibodies. Human brain tissues from neurologically diseased patients (n = 6 patients) were probed with (E) Bethyl Laboratories A300-660A, (F) Cayman Chemical, (G) Millipore, and (H) Cosmo Bio Co Ltd. Anti-PHLPP1.

**Figure 4:**
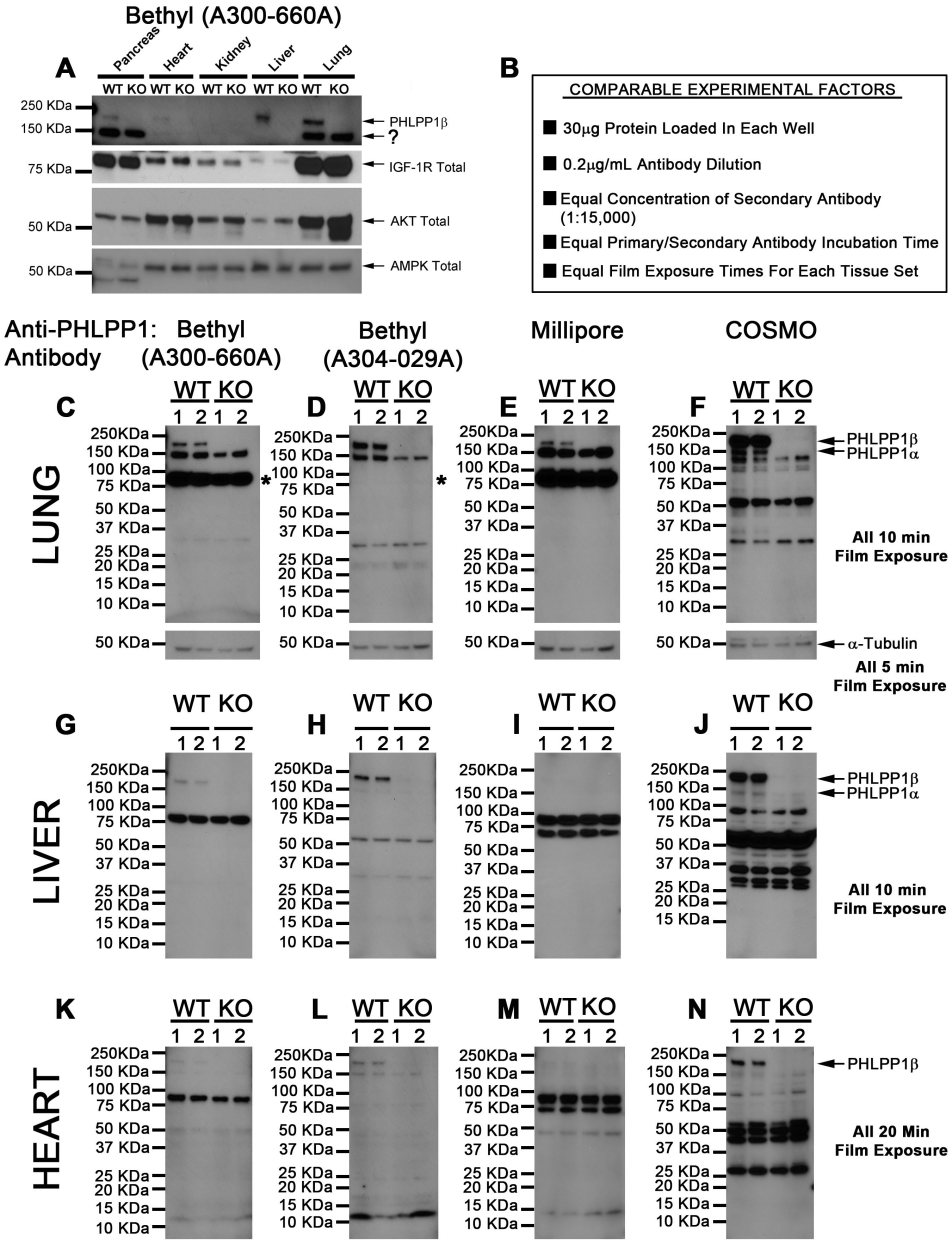
PHLPP1 Antibody Comparison in Peripheral Organs from WT and KO Mice. (A) Peripheral WT/KO tissues were harvested in order to screen other organs for potential interfering non-specific ~150 kDa bands. 20 µg/well pancreas, heart, kidney, liver, and lung were loaded onto SDS-PAGE, transferred to PVDF, and probed with Bethyl Laboratories A300-660A. (B) Lung, liver, and heart were selected for further analysis. Box shows experimental factors that were controlled to allow direct comparison of antibody specificity and selectivity. Of note, compared to brain, 10 fold higher antibody concentrations (0.2 µg/mL) were used to detect PHLPP1 proteins in peripheral tissues. (C–F) Lung tissues were probed for PHLPP1 using Bethyl Laboratories A300-660A, Bethyl Laboratories A304-029A, Millipore, and Cosmo, respectively. (G–J) Liver tissues were probed for PHLPP1 using Bethyl Laboratories A300-660A, Bethyl Laboratories A304-029A, Millipore, and Cosmo, respectively. (K–N) Heart tissues were probed for PHLPP1 using Bethyl Laboratories A300-660A, Bethyl Laboratories A304-029A, Millipore, and Cosmo, respectively. All blots show n = 2/genotype.

We next tested PHLPP1 antibodies in human derived cells/tissue. SHSY5Ys are neuronal-like cells which fully differentiate after 7d treatment with 10 μM retinoic acid (RA). We compared protein levels of PHLPP1 variants in undifferentiated (un-Diff) versus 7d RA differentiated (RA-Diff) neuronal cells. All antibodies detected ~190 kDa PHLPP1β in SHSY5Y homogenates, which appeared unaffected by differentiation (Figure 3A–4D). In contrast, changes in presumed PHLPP1α levels (i.e. false PHLPP1α is indicated by a question mark), are dependent on antibody selection. Bethyl Laboratories antibody (A300-600A) show that the ~150 kDa signal increased after RA differentiation (Figure 3A). Cayman Chemical antibody did not recognize a ~150 kDa signal or was below detection level (Figure 3B). Millipore antibody show that the ~150 kDa signal decreased after differentiation (Figure 3C). Finally, Cosmo antibody did not detect a 150 kDa signal (Figure 3D). We also compared PHLPP1 antibodies using human brain homogenates. Bethyl Laboratories (Figure 3E), Cayman Chemical (Figure 3F), and Millipore (Figure 3G) detected PHLPP1β and a strong ~150 kDa signal. Cosmo antibody detected PHLPP1β and a faint ~145–150 kDa signal (Figure 3G).

Confounding detection of non-specific ~150 kDa signal(s) may also be problematic in non-CNS tissues. Bethyl Laboratories antibody (A300-660A) was first used to screen peripheral organs including pancreas, heart, kidney, liver, and lung for the ~150 kDa non-specific band in WT/KO mice (Figure 4A). Insulin like growth factor 1 receptor (IGF-1R) total, AMP activated protein kinase (AMPK), and AKT total confirm that protein expression generally varies across organs (i.e. not unique to PHLPP1). We expanded investigation of PHLPP1 antibody comparisons in lung, liver, and heart (organs with high, medium, and low PHLPP1 expression, respectively). Experimental factors were controlled for those experiments (Figure 4B). Bethyl Laboratories (A300-660A), Bethyl Laboratories (A304-029A), Millipore, and Cosmo detected PHLPP1β in Lung, Liver, and Heart (Figure 4C–L and 4N) - except for Millipore/Heart which was below detection after 20 min exposure (Figure 4M). Bethyl Laboratories (A300-660A), Bethyl Laboratories (A304-029A), Millipore detect non-specific ~150 kDa protein in lung (Figure 4C–E). Only Cosmo detects bonafide PHLPP1α in lung and liver (Figure 4F and 4J, respectively). In our hands, α-Tubulin (i.e. loading control) was below detection in liver and heart. However, various non-specific bands detected by different PHLPP1 antibodies clearly show equal loading across samples.

## Discussion

PHLPP1 is abundant in brain^1^,^3^. Mounting evidence indicate it is a key regulator of neuronal survival as well as CNS function. Hippocampal PHLPP1β overexpression disrupts learning and memory in mice^4^. PHLPP1 gene deletion disrupts normal circadian rhythm in mice^9^. It also plays a detrimental role in brain injury. Synaptic PHLPP1 promotes neuronal excitotoxicity after injury^10^. It also inhibits protective AKT survival signaling in neurons^3^. Consistent with a detrimental role in brain, PHLPP1 KOs are protected against focal stroke^11^. Furthermore, hippocampal PHLPP1β increases after cardiac arrest in rat^12^. Thus it may alter brain recovery in different types of cerebral ischemia. Investigators continue to characterize the spatial, temporal, and relative expression levels of PHLPP1 in normal and pathological brain as well as other organs. Immuno-based proteomic techniques are widely utilized for such studies.

Here we report that PHLPP1 antibodies accurately detect PHLPP1β (~190 kDa) by Western analysis. In contrast, many commonly used antibodies do not accurately measure PHLPP1α levels (~145–150 kDa). Non-specific band(s) with similar molecular mass to PHLPP1α appear to hide the correct signal. A relatively new commercial PHLPP1 antibody, developed by the Kazusa DNA Research Institute (sold by Cosmo Bio Co Ltd.), accurately reports PHLPP1α levels by avoiding detection of overlapping erroneous ~150 kDa non-specific signals. To the best of our knowledge it has not been used in prior studies.

### Recommendations to Standardize PHLPP1 Western Blot Reporting

Review of PHLPP1 literature reveal continuing trends to exclude molecular weight standards in data presentation of Western blot figures - which may relate to article space/page restrictions. It is common to find a PHLPP1 figure showing a single cropped band labeled “PHLPP1” but lacking indication of estimated molecular mass (e.g.~145 kDa, ~150 kDa, and ~190 kDa) or named variant (α variant versus β variant). Those parameters should be acknowledged. Omitting that vital information make it difficult to retrospectively interpret results in light of our new findings. This is problematic because 1) PHLPP1α and PHLPP1β variants likely have different functions and roles in disease pathology, and 2) work here suggests that a dominant ~150 kDa non-specific signal(s) is preferentially recognized by many PHLPP1 antibodies – thus inaccurately describing PHLPP1α signaling changes.

Our results do not contest the validity of past studies using different PHLPP1 antibodies (especially if measuring PHLPP1β). Rather, we simply caution interpretation of the ~150 kDa band. If using other PHLPP1 antibodies not characterized here, we also recommend including knockdown control experiments to verify target specificity. Notably, some organs such as liver and heart do not appear to express high levels of non-specific ~150 kDa signal(s) – simplifying data interpretation in those tissues (PHLPP1β appears to be the primary signal). In contrast, the ~150 kDa non-specific signal is especially problematic in brain.

### Antibody Cross-Reactivity: A Challenge to Experimental Reproducibility

Antibody cross-reactivity remains a universal problem with immunoassays^13^,^14^,^15^,^16^. Antibody selection might strongly impact data interpretation - relevant to ongoing discussion concerning methods to enhance scientific reproducibility. We performed several simple experiments to demonstrate that PHLPP1 antibody selection can change data interpretation and fundamentally alter study conclusions. We first attempted to determine which PHLPP1 variant was highest in neurons versus astrocytes. Results using the Bethyl antibody suggest that PHLPP1α is highly abundant in neurons and almost absent in astrocytes (Figure 2A). Results using the Cosmo antibody suggest that PHLPP1α is absent in neurons with low expression in astrocytes (Figure 2B and 2C). Thus experimental conclusions/interpretations totally reverse depending on which antibody is used. We have discredited PHLPP1α results obtained by the Bethyl antibody (Figure 1 and Figure 4). However, it is hard to estimate the extent to which non-specific signal(s) have inadvertently been reported as PHLPP1 in past studies.

Tangentially, we do not know why PHLPP1α (as detected by Cosmo) has low expression in cortical neurons. Hippocampus appears to have relatively high levels of PHLPP1α. Future studies need to re-evaluate PHLPP1α signaling in different neuronal populations and glia. Levels may vary by cell type and location.

Our second experiment examined the effect of neuronal differentiation on PHLPP1 variants. Results of those studies also show that antibody selection dramatically changes data interpretation. The exact same cell homogenate samples were probed with four PHLPP1 antibodies. Bethyl Laboratories antibody revealed a ~150 kDa band increased after neuronal differentiation. Millipore antibody revealed a ~150 kDa band decreased after neuronal differentiation. Neither the Cosmo or Cayman antibodies detected a ~150 kDa band in SHSY5Y homogenates. The correct finding is most likely that PHLPP1α has low expression in SHSY5Y cells and is not altered by differentiation (i.e. Cosmo results). The ~150 kDa signals detected by Bethyl Laboratories/Millipore antibodies are likely non-specific proteins.

### Non-specific Signal(s)

The identity of potential cross-reacting ~150 kDa proteins detected by some PHLPP1 antibodies remain unclear. It may be a catenin related signal. A recent report combining immunoprecipitation and mass spectrometry identified a ~90 kDa β-catenin cross-reacting signal detected by some PHLPP1 antibodies^8^. Of note, Bethyl Laboratories responded to those concerns by discouraging use of product A300-660A for immunohistochemisy applications. They also generated a new PHLPP1 antibody with less reported β-catenin cross-reactivity (A304-029A)^17^. We verify that the new Bethyl Laboratories antibody does not detect β-catenin in brain, lung, liver, and heart. Nevertheless the improved antibody still appears to detect an erroneous PHLPP1α signal by Western blot analysis in PHLPP1 KO tissues.

Cross reactivity with PHLPP2, the second isoform of PHLPP, may be another explanation. PHLPP2 migrates at ~150 kDa by Western analysis^6^. Using Bethyl Laboratories antibodies we reported that a ~150 kDa PHLPP1α signal robustly increases with advancing developmental age in rat hippocampus^3^. In contrast, PHLPP2 antibody showed the opposite change – with advancing developmental age, PHLPP2 protein decreased in the same tissue homogenates. This suggests that ~150 kDa PHLPP1 signal(s), and ~150 kDa PHLPP2 signal, are distinctly different proteins. In addition, we found that PHLPP2 knockdown *in vitro* fails to decrease the ~150 kDa PHLPP1 signal (unpublished observations). Thus while it is reasonable to assume that the ~150 kDa PHLPP1 signal might be PHLPP2, we do not think it the explanation.

PHLPP1α lacks the large n-terminal exon 1 found in PHLPP1β (Ensemble Gene Code; ENSG00000081913). Lack of exon 1 is the only reported difference between PHLPP1 variants. Exon 4 was chosen for targeted disruption in PHLPP1 gene KO mice^9^. It is possible that the ~150 kDa band present in KO mice is a novel PHLPP1 variant which manages to avoid gene deletion. Such phenomenon has been described for other proteins. For instance, studies show that targeted disruption of exon 1 in the P2X7 gene fails to inhibit a functional protein variant in KO mice. This is due to an alternative start site downstream of the disrupted exon 1^18^. Exon 4 of the PHLPP1 gene is not predicted to be spliced or have an alternative downstream start site. Therefore targeted disruption of exon 4 should equally disturb both PHLPP1α and PHLPP1β protein expression. Consistent with that idea, PHLPP1α and PHLPP1β protein expression are absent in PHLPP1 KO brain/astrocytes as detect by Cosmo antibody (Figure 1F and 2C). Nevertheless, though unlikely, we cannot currently rule out the possibility that the ~150 kDa signal is a novel PHLPP1 variant that endogenously lacks exon 4 (thus evading deletion in KO mice but still detected by PHLPP1 antibodies). In summary, here we show that many PHLPP1 antibodies recognize a ~150 kDa signal in tissues that may represent a non-specific band unrelated to PHLPP1α.

## Methods

### Reagents

*PHLPP1 Antibodies:* Five anti-PHLPP1 antibodies were obtained from commercial sources. Publicly available manufacture information for each antibody is as follows: **(1)** Bethyl Laboratories Antibody (Cat# A300-660A; Lot#A300-600A-1, 9 amino acid (a.a.) antigen corresponding to the c-terminal side of human PHLPP1α/β; LPDYYDTPL, 1 mismatch to corresponding mouse sequence). **(2)** Bethyl Laboratories Antibody (Cat# A304-029A; antigen mapping somewhere between a.a. residues 1175-1225 of human PHLPP1. **(3)** Cayman Chemical Antibody (Cat#10007191; Lot#151571-151572 & Lot#04400591, 14 a.a. antigen corresponding to the c-terminal side of human PHLPP1α/β; YQLDQLPDYYDTPL, 4 mismatches to corresponding mouse sequence). **(4)** Millipore (Cat#07-1341; Lot#NG1820229, corresponding to the c-terminal side of human PHLPP1α/β). Alpha-Tubulin loading control was purchased from Cell Signaling technology. Goat anti-rabbit – HRP secondary was purchased from Life Technologies. **(5)** Cosmo Bio Co Ltd. (Cat#PRX-MKA0606AF, Lot#MKA0606AF[FAF01103], 118 a.a. sequence corresponding to the c-terminal side of mouse PHLPP1β: GSRVEVEVDIHCSRAKEKERQQHLLQVPAEASDEGIVISANEDESGLSKKADFSAVGTIGRRRAN-GSVAPQERSHNVIEVAADAPLRKPGGYFAAPAQPDPDDQFIIPPELEEEVKEI; 5 mismatches to corresponding human sequence.

### Animals

Methods were carried out in accordance with approved guidelines. All experiments were performed in accordance with relevant guidelines and regulations. All animal work was approved by the IACUC of the University of Pittsburgh. Euthanasia protocols follow recommendations established by the American Medical Veterinary Association Guideline for Euthanasia to minimize animal pain and suffering. Exon targeting gene deletion strategy to generate PHLPP1 KO mice was described by Masubuchi et al.^9^. Heterozygous PHLPP1 mice (+/−) on a B6/129 background were submitted to Jackson Laboratories for rederivation on a B61129SF1/J oocyte donor background. PHLPP1 +/+ and −/− mice were breed and genotyped at the Safar Center for Resuscitation Research. 10–15wk old male WT/KO mice were used for tissue collection and Western blot analysis. For PHLPP1 immunofluorescence on whole brain slices, a male KO (age 55wk) and female WT (age 39wk) were used. Female KOs were bred to male KOs to obtain all KO embryos for primary neuron and astrocyte culture. Female WT were bred to male WT to obtain WT cells for culture.

### Human Brain Tissue

Methods were carried out in accordance with approved guidelines. Informed consent was secured from all subjects in this study. Using a protocol approved by the University of Pittsburgh Medical Center IRB, Committee for Oversight of Research and Clinical Training Involving Decedents, de-identified human brain cortex samples (~20–40 mg each) were collected from three Alzheimer's and three neurologically diseased patients without Alzheimer, who died and samples subsequently stored at −80°C. Patient groups were comparable by age and gender. Tissue samples were homogenized according to procedures detailed below. Brain extracts were stored at −80°C until biochemical analysis.

### Ethics statement concerning animal work and collection of human tissues

The authors confirm that all experiments were performed in accordance with relevant guidelines and regulations. Animal work methods were carried out in accordance with procedures that were approved by the IACUC of the University of Pittsburgh. Methods of collection for human brain tissues were performed in accordance with procedures that were approved by an Institutional Review Board committee of the University of Pittsburgh Medical Center.

### Cell Culture

*Primary Neurons.* PHLPP1 WT and KO embryos were collected from timed pregnant females (E14–E16). Embryonic brains were isolated and meninges carefully removed under dissecting microscope. Cortical halves were separated and placed in ice cold hanks balanced salt solution (HBSS). Tissues were minced 1–2 min with sterile scissors in a 1.5 mL tube containing HBSS. Tissue was trypzinized for 8 min at 37°C, protease activity quenched with Neurobasal/B27 supplement + 10% fetal bovine serum (FBS), triturated ~10 times through a fire-polished glass Pasteur pipette, and dissociated cells counted on a hemacytometer. Neurons were seeded onto poly-D-lysine coated 6-well plates in Neurobasal/B27 culture media, and maintained by ½ media exchange every 3 day. ARA-C was added on day in vitro 3 (DIV) to prevent glia proliferation. Neurons were harvested for biochemistry on DIV6. *Primary Astrocytes*. Postnatal day (PND) 1-2 PHLPP1 WT and KO pups were collected and brains harvested. Meninges were carefully removed under dissecting microscope. Hemispheres dissociated by 10 min incubation in trypsin solution, passed through a 10 mL pipette 10-20X, and protease activity quenched in DMEM/F12/10%FBS (i.e. maintenance media). Total brain mix was seeded onto T75 flasks. Only astrocytes exponentially proliferate over culture days and serial passages. After 2-3 passages on T75 flasks, WT/KO astrocytes were prepared for downstream experiments and seeded onto 6-well plates. *Human neuronal SHSY5Ys*. Cells were purchased from ATCC. Undifferentiated SHSY5Ys were propagated in Optim-MEM culture media with 10%FBS on T75 culture flasks. Cells were trypsinized and seeded onto 6 well plates. Undifferentiated cells were collected for biochemistry 3d after plating. SHSY5Ys were differentiated over 7d by media replacement with Opti-MEM containing 10 μM RA + 1%FBS. Differentiated neurons were harvest for biochemistry on day 7.

### Western Blot Analysis

Tissues and cells were homogenized in RIPA buffer containing protease inhibitors, phosphatase inhibitors and EDTA. Samples were pulse-sonicated for 20–30s. Protein concentrations were analyzed using the BCA assay. Samples were prepared in Laemmli loading buffer. Protein samples were loaded onto gradient TGX precast SDS-PAGE gels (BioRad): 20 μg/well for human brain tissue, 30 μg/well for mouse WT/KO tissues, 20 μg/well for human neuronal SHSY5Y electrophoresis experiments, and 10–20 μg/well for primary neuron and astrocyte studies. In the latter primary neuron versus astrocyte study, total protein stain was visualized using reversible Swift Membrane Stain (Fisher Scientific). Proteins were electrophoresed at ~150 V for ~1 h. Proteins were transferred to PVDF membranes (100 V/1.15 h/4°C). Blots were blocked 1 h in tris-buffered saline with tween-20 (TBST) + 7.5% milk. Primary antibodies were prepared in TBST/milk and incubated overnight on a rocker at 4°C. Blots were washed 3X in TBS, incubated 2 h in secondary antibody (1:15,000), washed 3X in TBS, incubated 2 min with HRP detection reagent (PIERCE), and films exposed/developed in a dark room. Antibodies were individually optimized for detection of PHLPP1 except for Figure 1 and Figure 4 – antibody optimization parameters are equivalent in those studies. Films were captured on a 600–1200 dpi flatbed scanner and images compiled in Photoshop.

### Immunofluorescence

*In Vitro Neuron Culture*: Sterile 8-well chamber glass culture slides were treated with poly-D-lysine overnight at 37°C. WT and KO neurons were seeded onto glass slides. DIV8 neurons were washed with PBS, fixed ~25 min with 4% paraformaldehyde, washed with PBS, cell membrane permeabilized with 0.1% Trition X-100/PBS, washed with PBS, blocked ~45 min with 20% goat serum + 1%BSA in PBS, and incubated overnight at 4°C with primary antibody dissolved in 3% goat serum/PBS. Neuronal β-tubulin III (neuron marker) was purchased from abcam. Wells were washed with PBS, incubated with Alexa Fluor Goat-anti Rabbit 594 and Alexa Fluor Goat-anti mouse 488 (Life Technologies) for ~1.5 h. Wells were washed with PBS, plastic chamber removed, and glass slide mounted with ProLong Gold Antifade with DAPI (Life Technologies). Images were collected (20× magnification) on a fluorescent microscope (Eclipse 50 Nikon, Melville, NY, USA) and complied in Photoshop. *Ex Vivo Brain Sections*: WT and KO mice were anesthetized, transcardially perfused with PBS followed by 10% formalin. Brains were collected and post-fixed in 10% formalin followed by 30% sucrose solution. Brains were sectioned, mounted on glass slides, and incubated with primary rabbit anti-PHLPP1 antibody (Cosmo) as well as either mouse anti-neuronal tubulin III (Neuron Marker) or GFAP (astrocyte marker). Sections were washed and incubated with Alexa Fluor Goat-anti Rabbit 594 (RED) and Alexa Fluor Goat-anti mouse 488 (GREEN). 20× images were collected on a fluorescent microscope.
